# Aphids Are Unable to Ingest Phloem Sap from the Peduncles of Lime Fruits

**DOI:** 10.3390/plants9111528

**Published:** 2020-11-10

**Authors:** Carolina Vázquez, Michele Carmo-Sousa, Joao Roberto Spotti Lopes, Alberto Fereres, Aranzazu Moreno

**Affiliations:** 1 Instituto de Ciencias Agrarias, Consejo Superior de Investigaciones Científicas, ICA-CSIC, 28006 Madrid, Spain; carolinaeloisavc@gmail.com; 2 Department of Entomology and Acarology, Luiz de Queiroz College of Agriculture, University of São Paulo, Piracicaba SP 13418-900, Brazil; m.sousatimossi@gmail.com (M.C.-S.); jrslopes@usp.br (J.R.S.L.)

**Keywords:** CTV, transmission, *Aphis (Toxoptera) citricidus*, *Aphis gossypii*, electrical penetration graph, peduncles

## Abstract

Citrus exports to Europe are regulated enforcing that fruits shall be free from peduncles and leaves, as they represent an important pathway for the entrance of non-European (non-EU) *Citrus tristeza virus* (CTV) isolates into the European Community. Aphids, are the vectors of CTV and could potentially feed on peduncles of imported fruits and thus spread non-EU isolates of CTV across Europe. We studied the probing behaviour of the main vectors of CTV (*Aphis* (*Toxoptera*) *citricidus* and *Aphis gossypii*) on lime leaves and peduncles to assess whether they could potentially transmit the virus. Aphids placed on peduncles rejected probing and feeding, tried to escape and spent most of their time on non-probing activities. Our work demonstrated that both *A. citricidus* and *A. gossypii* could not ingest sap from the phloem of lime peduncles, as phloem ingestion was never observed. This implies that aphids would not be able to acquire CTV from an infected fruit peduncle and transmit it to a susceptible plant. Our study supports that citrus exports with fruit peduncles to Europe may not be a real risk for the introduction of non-EU isolates of CTV to the European Community.

## 1. Introduction

Citrus is one of the most important crops of agricultural systems worldwide, at more than 100 million tons of production in 2008 [1], with its wide range of varieties providing employment and commerce. In fact, the value of world fruit and vegetable exports was US$ 34.6 billion in 2001, where fruit accounted for almost 60% of this and the main percentage (21%) was represented by citrus, followed by bananas and grapes [2]. However, citrus production is threatened by abiotic factors (e.g., droughts) and biotic factors (especially pests and diseases) that have negative effects on the growth, development and yield of the crop. Among the biotic factors, “Huanglongbing” (HLB), caused by phytopathogenic bacteria (*Candidatus* Liberibacter spp.), and “Tristeza”, caused by *Citrus tristeza virus* (CTV), are two of the most devastating and harmful diseases. “Tristeza” is the main viral disease of citrus, causing the death of almost 100 million trees grafted onto sour orange (*Citrus aurantium*) rootstock [3].

CTV is a well-characterized virus in the genus *Closterovirus* of the Closteroviridae family [4,5] that is restricted to phloem tissue. It has a single-strand positive RNA genome that consists of ~19,296 nucleotides, the largest genome among RNA plant viruses [6], and it is characterized by the genetic diversity of its isolates. These biological properties apply to all CTV isolates described currently, although different CTV isolates can cause different symptoms and can differ in their vector transmission properties. Some genome sequences of European CTV isolates are well studied [7], and these studies show that several CTV isolates/strains (e.g., RB isolates) are not known to occur in Europe. Even if sequence variants genetically similar to some non-European (non-EU) CTV isolates have been detected in the EU [8], the symptoms have not been observed in field surveys. CTV has been reported in most citrus-growing areas of all five continents. In Europe, CTV is present in seven out of the eight EU member states with significant citrus production (Spain, Italy, Portugal, Greece, France, Croatia and Cyprus) [9], but it was also reported in other EPPO regions, such as Albania, Bosnia and Herzegovina, Georgia, Montenegro or Turkey [10]. However, country reports in general do not specify the presence of particular CTV isolates.

There are three major disease syndromes associated with CTV: ‘Tristeza’, which is a decline that leads to wilting until the death of a tree; stem pitting (SP), a syndrome characterized by the development of pits in the trunk and stems of a tree that leads to loss of plant vigour, dwarfing, severe yield reduction and small fruit size; and seedling yellow (SY), which occurs normally in greenhouses, where it affects young plants by producing yellowing and stunting [11,12,13]. These three syndromes in citrus can be severe or mild, but CTV may also remain completely latent. The severity of symptoms depends on the nature of the virus strain, host plant species, rootstock/scion combination, and the presence of stress factors [12].

CTV is a graft-transmissible agent, which means that it can be transmitted through vegetative replication of infected host plants, and this process is responsible for most CTV introductions into new areas. CTV is also naturally transmitted by several aphid species [12,14] in a foregut-borne, semipersistent manner [15], and this type of virus propagation is important for local spread. Semipersistent viruses are a group of non-circulative viruses that are restricted to the phloem and are retained for several hours by their aphid vectors [16]. CTV aphid transmission is affected by several factors, such as the differences among the isolate/strain of the virus, plant donor and receptor varieties, environmental conditions, and number and species of aphids involved in the processes [17]. The most efficient vector of CTV, *Aphis* (*Toxoptera) citricidus* (Kirkaldy) [12,18], also known as the brown citrus aphid, can spread especially severe CTV strains, which other vectors cannot transmit easily [19]. Within Europe, *A. citricidus* is only reported in Portugal and Spain but away from the main citrus production areas [10]. In the Mediterranean basin citrus growing areas, where *A. citricidus* is absent, *Aphis gossypii* (Glover) is the main CTV vector, playing a major role in virus spread in countries such as Spain and Israel [20,21,22].

Accordingly, the export and movement of plant material are designed to prevent virus spread. Exports to the European Union are regulated by Council Directive 2000/29/EC [23] on protective measures against the introduction of organisms harmful to plants or plants products into the community and against their spread within the community, and this measure has now been reapplied by Regulation (EU) 2016/2031 [24] on protective measures against pests of plants. In 2017, the EFSA [25] provided a pest categorization of harmful organisms, concluding that CTV “*has had and will have a very considerable impact on the EU citrus industry*” due to the non-EU CTV isolates that produce stem pitting (SP) on lime, grapefruit and sweet orange, “*a syndrome against which the European orchards are not protected*”. Therefore, Council Directive 2000/29/EC [23] includes non-EU CTV isolates (defined by their geographical origin outside of the European Union territory) as a Union quarantine pest. Therefore, legislation establishes a special requirement for that group of pests, which must be implemented by all member states for the introduction and movement of plants, plant products and other objects into and within all member states. The legislation states that fruits of *Citrus* L., *Fortunella* Swingle, *Poncirus* Raf., and their hybrids originating outside the Community, “*shall be free from peduncles and leaves, and the packaging shall bear an appropriate origin mark*” [23].

Studying the probing and feeding behaviour of the main aphid vectors is important to understand the transmissibility and the management of viral diseases such as CTV. The electrical penetration graph (EPG) technique has been widely applied to investigate insect-plant-pathogen interactions [26], including the probing and feeding behaviour activities of sap-sucking insects associated with the transmission of plant pathogens by their insect vectors [15]. EPG allows the study of vector probing and feeding behaviour based on the analysis of electrical waveforms generated as an insect penetrates its stylets through various plant tissues [27,28].

The transmission of phloem-limited viruses, such as CTV, is dependent on aphid stylet activities in phloem tissues [29]. Previous EPG studies showed that some semipersistent phloem-restricted virus species, such as the crinivirus *Lettuce chlorosis virus* (LCV), transmitted by the whitefly *Bemisia tabaci* (Gennadius), are inoculated primarily during phloem salivation (waveform E1), while its acquisition occurs only during the phloem ingestion phase (waveform E2) [30]. The same occurs for the waikavirus *Maize chlorotic dwarf virus* (MCDV) transmitted by the black-faced leafhopper *Graminella nigrifrons* (Forbes) [31]. A recent study reporting the probing activities of the aphid *Myzus persicae* (Sulzer) related to the semipersistent transmission of the closterovirus *Beet yellows virus* (BYV) to sugar beet showed for the first time that virus inoculation occurs during specific brief intracellular stylet punctures in the phloem tissues (called phloem-pds) and before the phloem salivation phase (waveform E1) [32]. However, *M*. *persicae* did not acquire BYV from the infected-source plant before reaching the phloem salivation phase. 

Not only the duration of acquisition access periods but also the concentration of virions in source plants are two factors that determine the transmission of phloem-limited viruses by their aphid vectors [33]. The study conducted by Bertolini et al. [34] reported a higher CTV number of copies in fruit peduncles than in young and mature shoots. This could enhance the risk of acquisition and subsequent transmission of the virus when aphids feed on the peduncle of infected fruits. Despite the high efficiency of *A. citricidus* in transmitting non-EU CTV isolates [19,22,35], it is still unknown whether aphids can acquire and transmit CTV when probing on fruit peduncles. Furthermore, the main vector species of CTV in Spain, *A. gossypii* [20], could potentially acquire and transmit the virus when probing on peduncles of citrus fruits. Therefore, it is important to clarify whether aphids can feed on fruit peduncles and whether the presence of peduncles in imported fruits constitutes a real risk for entry of non-EU CTV isolates into the European Community.

Hence, the objective of this study was to investigate the probing and feeding behaviour activities of the most important aphid vectors of CTV when exposed to leaves and peduncles of Tahiti lime to determine whether the vector species can ingest phloem sap and therefore acquire this phloem-restricted virus from leaves and fruit peduncles.

## 2. Results

### 2.1. Probing Behaviour of Aphis citricidus on Fruit Peduncles and Lime Leaves

The probing and feeding behaviour of a total of 29 individual adults of *A. citricidus* (15 on lime peduncles and 14 on lime leaves) during the 8 h of the EPG were analysed. The results of the proportion of aphids that performed each type of waveform are shown in Table 1. All insects started stylet pathway activities (C waveform—intercellular apoplastic stylet pathway) regardless of treatment (fruit peduncles or lime leaves). However, significant differences were observed in the probing and feeding behaviour in phloem tissues between treatments: the proportion of aphids reaching and probing from the phloem (E1 and E2) on the fruit peduncles was significantly lower than that on the lime leaves. None of the aphids probing on fruit peduncles were able to reach the phloem sieve elements (Proportion of individuals that produce E1: 0/15).

A comparison of the analysed EPG behavioural variables of *A. citricidus* is shown in Table 2. Significant differences in the probing behaviour of *A. citricidus* exposed to fruit peduncles and lime leaves were obtained. A higher number of intercellular apoplastic stylet probes (number of C waveforms within the probes) was observed in insects exposed to the fruit peduncle (number of C: 10.73 ± 2.11) than those exposed to lime leaves (number of C: 3.50 ± 0.49) (*p* = 0.0007). Significant differences were also obtained in the number of waveforms related to phloem activities (salivation in phloem tissues—E1; phloem sap ingestion—E2) and no phloem contacts were observed for aphids probing on fruit peduncles (*p* = 0.0032).

When the mean waveform duration per insect was evaluated, the duration of the stylet pathway phase (C) was longer for insects that were probing on leaves (total C duration: 159.57 ± 35.16 min) than those probing on peduncles (total C duration: 99.91 ± 21.57 min), but no significant differences were observed (*p* = 0.1761) (Table 2). It was also observed that the aphids remained for a longer period (almost 3 times more) conducting non-probing activities (np waveform—walking or resting) on the fruit peduncles (total np duration: 376.93 ± 20.87 min) than on the lime leaves (total np duration: 126.54 ± 35.69 min) (*p* < 0.0001) (Table 2), which significantly reduced the chance of phloem-related activities.

No phloem activities were observed for *A. citricidus* probing on fruit peduncles for 8 h, resulting in significant differences for both the total E1 and E2 durations, when compared with aphids probing on lime leaves. Aphids probing on leaves were able to reach the phloem and ingest phloem sap for more than 3 h on average (total E2 duration: 193.31 ± 29.16 min) (*p* = 0. 003) (Table 2).

The percentage of time that aphids spent conducting each probing activity (or waveform event) is represented in Table 3. When *A. citricidus* was placed on fruit peduncles, the percentage of the total time spent on non-probing events (np) was much longer (78.53%) when compared with that on lime leaves (26.36%; *p* < 0.0001), but the percentage of time spent on phloem ingestion (E2) was significantly (*p* < 0.0001) lower on peduncles (0%) than on lime leaves (40.27%). No differences were found between treatments for the non-phloem phase or stylet pathway activities (waveform C) (*p* = 0.585) and salivation in phloem sieve elements (E1) (*p* > 0.999).

### 2.2. Probing Behaviour of Aphis gossypii on Fruit Peduncles and Lime Leaves

The probing behaviour of 32 individual adult *A. gossypii* (16 on fruit peduncles and 16 on lime leaves) were analysed. The results of the proportion of aphids that performed each type of waveform are shown in Table 1. No significant differences were found in the proportion of *A. gossypii* individuals able to probe peduncles and lime leaves (proportion of individuals that produce C was 16/16 in both fruit peduncles and leaves). The analysis of the probing and feeding behaviour of *A. gossypii* (number and duration of the EPG waveforms) showed that there were striking differences between aphids placed on fruit peduncles and those probing on lime leaves (Table 2). A delayed start to probing was observed when *A. gossypii* aphids were exposed to fruit peduncles (start EPG—1st Probe: 3.57 ± 1.36 min) when compared with those exposed to lime leaves (start EPG—1st Probe: 19.83 ± 2.32 min) (*p* < 0.0001) (Table 2). Once insects started the probing activities, the differences were not significant in the number and duration of intercellular apoplastic stylet pathway (C) between aphids probing on fruit peduncles (number of C: 14.50 ± 2.09; Total C duration: 187.16 ± 18.00 min; Mean duration of C events: 15.51 ± 2.09 min) and those probing on lime leaves (number of C: 14.13 ± 1.98; Total C duration: 169.94 ± 25.57 min; Mean duration of C events: 15.30 ± 4.59 min) (number of C *p* = 0.844; Total C duration *p* = 0.586; Mean duration of C events *p* = 0.575).

The results obtained for *A. gossypii* also showed clear differences for most of the phloem-related activities, which are directly involved in the acquisition and inoculation of CTV (Table 1 and Table 2). The proportion of *A. gossypii* that reached the phloem sieve elements on fruit peduncles was significantly lower than that on lime leaves (*p* = 0.0001). Only a single recording showed an E1 waveform in the fruit peduncles (proportion of individuals that produce E1 = 1/16), with a total duration of 261.62 s, while 15 recordings showed an E1 waveform with a total duration of 7617.93 s in lime leaves. Moreover, the single aphid that was able to reach the phloem sieve elements in the peduncle was unable to ingest phloem sap (no E2 phase was observed; Proportion of individuals that produce E2 = 0/16). Conversely, all aphids probing on lime leaves that reached the phloem tissues were able to ingest phloem sap (proportion of individuals that produce E1 = 15/16 and Proportion of individuals that produce E2 = 15/16) (*p* = 0.0001).

When aphids probed on fruit peduncles, the number (number of E1 per insect: 0.13 ± 0.13) and total duration (total E1 duration: 0.27 ± 0.27 min) of the phloem salivation phase were significantly reduced when compared to those of aphids probing on lime leaves (number of E1: 3.31 ± 0.74; Total E1 duration: 7.93 ± 2.27 min) (number of E1 *p* < 0.0001; Total E1 duration *p* < 0.0001). Additionally, the time to reach the phloem sieve elements was significantly (*p* < 0.0001) longer in aphids probing on fruit peduncles (start EPG—1st E: 463.61 ± 16.38 min) than in those probing on lime leaves (start EPG—1st E: 175.84 ± 34.26 min).

The percentage of each stylet activity or waveform event is represented in Table 3. Similar to that for *A. citricidus*, when *A. gossypii* was exposed to fruit peduncles, the percentage of the total time spent in non-probing events (np) was higher (60.95%) than that in those exposed to lime leaves (20.22%; *p* < 0.0001). However, the percentage of time spent in phloem-related activities (E1 and E2) was much lower on fruit peduncles than on the leaves, although no differences were found between treatments for salivation into phloem sieve elements. The percentage of time spent ingesting phloem sap was significantly lower in peduncles (0%) than in lime leaves (33.21 %; *p* < 0.0001).

## 3. Discussion

CTV is a phloem-limited virus that affects citrus orchards worldwide, causing overwhelming effects in plants and yield reduction [20,36]. The role played by the different CTV strains and their potential introduction into European countries not yet affected has led to the banning of the export of citrus fruits to the European community that contain leaves and peduncles [25], as mentioned before. Although imported fruits may be a source of CTV for aphid transmission if leaves are present, it is still unclear whether aphids can ingest phloem sap and acquire the virus from fruits bearing only peduncles.

*Aphis gossypii* is the main vector responsible for the spread of CTV [20] and the most likely vector of CTV in Europe. In Brazil, the most efficient vector is *A. citricidus* [37], which can spread especially severe CTV strains [19]. Our results obtained for both *A. gossypii* and *A. citricidus* have practical implications because most plant pathogens rely on vectors to spread to new areas, and the probing behaviour of these vectors are strongly correlated with the epidemiology of these pathogens. We found that aphids exposed to lime leaves had probing and feeding behaviour similar to those expected under optimal feeding conditions, which means that lime leaves are excellent feeding sources for both aphid species. Additionally, when aphids fed on leaves, they managed to reach the phloem promptly and initiate salivation in the phloem sieve elements followed by phloem sap ingestion for extended periods of time. This particular behaviour is known to facilitate both the acquisition and inoculation of phloem-restricted closteroviruses [38].

Conversely, aphids were able to start probing activities immediately when exposed to leaves or peduncles, but the percentage of time spent on non-probing activities was significantly higher on fruit peduncles than on lime leaves. Furthermore, the main CTV vectors rejected ingestion from the phloem of fruit peduncles under our experimental conditions. It is important to highlight that aphid movement is very limited when connected to an EPG probe because they are tethered to a very short gold wire (2 cm in length). Therefore, under natural and free choice conditions, aphids that land on a fruit peduncle will unlikely initiate probing will reject feeding and will be unable to acquire any phloem-restricted viruses that might be present on the fruit peduncle.

Moreover, phloem-related activities were rarely observed when both aphid species were placed in peduncles; they spent the vast majority of the time in intercellular apoplastic stylet pathway C or on non-probing activities (walking and resting). We hypothesize that vascular tissues of lime peduncles are lignified producing thick cell walls of sclereids that very likely prevented the penetration of aphid stylets into the phloem sieve elements. This type of tissue modifications to cope with increasing fruit weights have been reported for apple fruit peduncles [39].

Phloem-related activities are important to highlight in this study due to the semipersistent characteristic of CTV. The semipersistent virus category includes noncirculative viruses that have a retention time of several hours in their vectors and are often restricted to the phloem of the infected plant. Therefore, longer acquisition and inoculation access periods are needed to transmit them [16]. As CTV is a phloem-restricted virus [40], aphids must reach the phloem to release the virus particles and inoculate a healthy plant either with egested food and/or watery saliva excreted during the phloem salivation phase (E1 waveform) [41]. Approximately 2–3 h on average is the time required by aphids to reach the phloem sieve elements [16]. Campolo [42] observed that *A. gossypii* was able to transmit the virus to healthy Mexican lime plants after 60 min, but in our study, aphids exposed to fruit peduncles were unable to reach the phloem during the 8 h of recording. In fact, *A. citricidus* never reached the phloem tissues when probed in peduncles, and only a single individual of *A. gossypii* was able to reach the phloem and started the phloem salivation phase in this part of the plant. Hence, the sharp reduction observed in the number and duration of phloem salivation activities in peduncles will significantly reduce the chances of CTV transmission. Nevertheless, recent work has shown that other semipersistent viruses, such as Beet yellows virus (BYV; *Closterovirus*), are inoculated during brief intracellular punctures in phloem cells just before the phloem salivation phase (waveform E1) [38,43], but this is still unknown to occur with CTV. This is important to consider because both *A. citricidus* and *A. gossypii* could inoculate a phloem-restricted virus during specific intracellular stylet punctures (phloem-pds) in either sieve elements or companion cells.

It is known that after the phloem salivation phase of aphids, passive phloem ingestion (E2 waveform) usually starts and lasts from a few min to several hours. Phloem sieve elements have a very high positive pressure, which makes sap ingestion occur passively. To acquire the CTV virions, aphids must ingest phloem sap from the sieve elements (E2 waveform), and then virions, need to adhere to the aphid’s foregut [44]. In the same study mentioned previously, Campolo [42] reported that *A. gossypii* was able to acquire CTV when probing for 30 min on infected Madame Vinous plants. Our results show that although a single *A. gossypii* was able to reach the phloem and started the phloem salivation phase when probed in peduncles, it was unable to ingest from the sieve elements. This result has practical implications in relation to the transmission of aphid-vector, phloem-restricted, semipersistent viruses, which are exclusively acquired during phloem sap ingestion [38]. Therefore, the acquisition of CTV will be abolished when aphids are exposed to fruit peduncles.

Despite the high concentration of CTV virus particles found in fruit peduncles [34,45], the transmission will unlikely occur due to the preferences of the aphid vectors for probing in another part of the plant, the difficulties that aphids have in reaching the phloem and their inability to ingest phloem sap when exposed to lime fruits. Accordingly, the acquisition of CTV from lime fruits infected by aphids will not be possible based on the results of the probing behaviour of both *A. citricidus* and *A. gossypii*.

In conclusion, the present study on the probing and feeding behaviour of the main CTV vectors indicates that no virus transmission would occur following aphid exposure to fruit peduncles. Both *A. citricidus* and *A. gossypii* exposed to fruit peduncles were unable to ingest phloem sap as opposed to those exposed to lime leaves. Hence, our study supports that citrus exports with fruit peduncles may not be a real risk for the introduction of non-EU isolates of CTV to the European community.

## 4. Materials and Methods

### 4.1. Aphid Rearing and Test Plants

Healthy apterous adults of two aphid CTV-vector species, *A. citricidus* and *A. gossypii,* were used in this study. A colony of *A. citricidus* was originally collected from citrus in Piracicaba (São Paulo State -SP), Brazil, in 2019 and reared on caged, healthy sweet orange seedlings [*Citrus sinensis* (L.) Osbeck] cv. “Hamlin”, under controlled conditions in a growth chamber at 25 ± 2 °C and photoperiod of 14:10 h (L/D), at the University of São Paulo (Piracicaba, SP). *A. gossypii* was collected in 2011 at Moncada (Valencia, Spain) on tangerine (*Citrus reticulata* Blanco) trees. The *A. gossypii* colony was reared at ICA-CSIC facilities on three-week-old cotton plants (*Gossypium hirsutum* L. var. *Deltapine*-61) to simplify rearing and regular maintenance tasks. At ICA-CSIC, the aphids were reared inside rearing cages with a fine net to allow ventilation under controlled conditions in a “walk-in” chamber at a temperature of 23:18 °C (D/N) and a photoperiod of 16:8 h (L/D).

Plants used for the EPG experiments were healthy Tahiti lime (*Citrus latifolia*) in both Spain and Brazil (vegetative stage 32–39 according to BBCH scale). Plants were maintained in a greenhouse at a temperature of 25:20 °C ± 1 °C (D/N) and a photoperiod of 16:8 h (L/D) in Spain and Brazil, and the plants were kept in a temperature-controlled greenhouse (25 ± 5 °C).

### 4.2. Stylet Activities of Aphis citricidus and Aphis gossypii on Lime Leaves and Fruit Peduncles

To study the stylet activities associated with the transmission of *Citrus tristeza virus* by their aphid vectors, *A. citricidus* and *A. gossypii,* their probing and feeding behaviour on fruit peduncles and young shoot leaves of lime were monitored using the electrical penetration graph (EPG) technique [46].

Apterous adult aphids were immobilized by a vacuum and attached to a gold wire (2 cm length, 18.5 µm diameter)—previously attached to a copper electrode measuring 2 cm length)—by the dorsum using hand-mixed, water-based silver conductive paint glue (EPG System, Wageningen, the Netherlands). Then, the electrode was inserted into the input of the EPG probe, and to complete the electrical circuit, another copper electrode (10 cm length, 2 mm diameter) was inserted into the potting substrate (for the lime plants) or directly into the fruit (for the peduncles). Aphids were placed on the abaxial side of new leaves or fruit peduncles of the limes and allowed to probe and feed for 8 h.

The probing and feeding behaviour of each aphid was monitored by Giga-8 or Giga-4 direct current-EPG (DC-EPG) devices with 1 GΩ of resistance (EPG Systems, Wageningen, Güeldres, The Netherlands) [27,47]. A USB analogue/digital converter card (DI-155 and DI-710; DATAQ Instruments, Akron, OH, USA, EEUU) was used to transfer the EPG signals to a PC. The monitoring system was placed in a Faraday cage to avoid external noise in an air-conditioned room (25 ± 2 °C) with artificial light provided by fluorescent lamps (the same conditions in Spain and Brazil). A minimum of 14 recordings were made for each treatment, and a different single aphid and plant/fruit was used for each replicate.

EPG signals were acquired and analysed using Stylet+ software for Windows (EPG Systems). EPG variables were processed using the EPG-Excel Data Workbook developed by Sarriá et al. [48] (2009). The EPG waveforms associated with specific stylet tip positions and activities when insects probed and fed on the different tissues of lime were as follows: waveform np, which represents non-probing behaviour (no stylet contact with the leaf/peduncle tissue), and waveform C, which represents the intercellular apoplastic stylet pathway where the insects show the cyclic activity of mechanical stylet penetration and secretion of saliva. Two waveforms related to phloem activity were recorded: waveform E1, which represents salivation into phloem sieve elements at the beginning of the phloem phase, and waveform E2, which is correlated with passive phloem sap uptake from the sieve element. Furthermore, waveform G, which represents active intake of xylem sap, and waveform F, which is related to derailed stylet mechanics were also recorded.

To compare the probing behaviour of *A. citricidus* and *A. gossypii* on the different lime leaves and peduncles, a selected set of EPG variables was calculated as follows: Proportion of individuals that produced a specific waveform type (number of insects that produce a specific waveform divided by the total number of insects for each treatment), number of waveform events for each insect (the number of times that a waveform occurs for each insect), total waveform duration for each insect (the total duration of a waveform, summed over all occurrences of the waveform for each insect) and the mean duration of waveform events for each insect (the mean waveform event duration (total waveform duration divided by number of waveform events) for each insect). The output given by the Sarriá et al. [48] (2009) workbook for each given insect (replicate) was used to calculate the treatment mean for each variable.

Then, the data of the total duration of the waveforms were used to calculate the mean percentage of time spent on each stylet activity (np, non-phloem phase; E1; E2) per treatment.

### 4.3. Statistical Analysis

The two aphid species were studied independently. The raw data of *A. citricidus* and *A. gossypii* were checked for normality and homogeneity of variance using Shapiro–Wilk W and were transformed with sqrt (x + 1) and ln (x + 1) if needed to reduce heteroscedasticity. Most EPG variables did not follow a normal distribution; therefore, they were compared by the non-parametric Mann–Whitney U-test or Student’s *t*-test for parametric variables at a 0.05 significance level using SPSS V26.0 Statistics software (IBM^®^) for *A. gossypii* data. Additionally, the comparisons between the proportions of individuals who produced a given type of wave were analysed using the chi-square test (*χ*^2^). The mean percentage of time spent on each waveform for each treatment was compared with the chi-square test (*χ*^2^) using StatView 4.01 SE + Graphics.

## Figures and Tables

**Table 1:** Proportions of aphids that showed a particular waveform on fruit peduncles and young leave shoots of limes in 8 h of EPG recording of *A. citricidus* and *A. gossypii*. Highlighted in bold those variables that showed striking significant differences between treatments.

Aphid Species	Waveforms *	Fruit Peduncles	Lime Leaves	*χ* ^2^	*p*
*Aphis citricidus*	C	100 (15/15) a	100 (14/14) a	-	-
	E1	0 (0/15) b	64.3 (9/14) a	13.982	**0.0002**
	E2	0 (0/15) b	64.3 (9/14) a	13.982	**0.0002**
*Aphis gossypii*	C	100 (16/16) a	100 (16/16) a	-	-
	E1	6.25 (1/16) b	93.75 (15/16) a	24.500	**0.0001**
	E2	0 (0/16) b	93.75 (15/16) a	28.240	**0.0001**

* **Waveforms**: (C) stylet pathway activities, (E1) salivation in phloem sieve tubes, (E2) phloem sap ingestion. a, b: Proportions followed by the same letter, in the same row, do not differ significantly (*p* > 0.05) according to the chi-square (*χ*^2^) test.

**Table 2:** EPG variable values (means ± standard error) during the probing and feeding behaviour of *A. citricidus* and *A. gossypii* on fruit peduncles and leaves of Tahiti lime during an 8-h recording. Highlighted in bold those variables that showed striking significant differences between treatments.

Aphid Species	EPG Variables	Fruit Peduncles	Lime Leaves	*p*
*Aphis citricidus*	Number of Waveform Events			
	np	11.60 ± 2.07 a	3.29 ± 0.49 b	**<0.0001**
	C	10.73 ± 2.11 a	3.50 ± 0.49 b	**0.0007**
	E1	0 ± 0 b	0.93 ± 0.25 a	**0.0032**
	E2	0 ± 0 b	0.93 ± 0.25 a	**0.0032**
	Total Waveform Duration (min)			
	np	376.93 ± 20.87 a	126.54 ± 35.69 b	**<0.0001**
	C	99.91 ± 21.57 a	159.57 ± 35.16 a	0.1761
	E1	0 ± 0 b	0.57 ± 0.15 a	**0.0032**
	E2	0 ± 0 b	193.31 ± 49.16 a	**0.0032**
	Mean Duration of Waveform Events (min)			
	np	32.49 ± 6.04 a	30.26 ± 8.69 a	0.696
	C	9.31 ± 0.98 a	45.59 ± 11.31 b	**<0.0001**
	E1	0 ± 0 a	0.62 ± 0.05 b	**<0.0001**
	E2	0 ± 0 a	208.18 ± 45.95 b	**<0.0001**
	Sequential ^a^ (min)			
	Start EPG—1st Probe	8.30 ± 4.78 a	10.51 ± 3.29 a	0.2752
	Start EPG—1st E	480.00 ± 0.00 a	225.67 ± 53.84 b	**0.0094**
	Number of Waveform Events			
	np	15.06 ± 2.13 a	11.63 ± 1.59 a	0.178
	C	14.50 ± 2.09 a	14.13 ± 1.98 a	0.844
	E1	0.13 ± 0.13 a	3.31 ± 0.74 b	**<0.0001**
	E2	0 ± 0 a	3.00 ± 0.68 b	**<0.0001**
	Total Waveform Duration (min)			
	np	292.57 ± 17.99 a	93.31 ± 21.07 b	**<0.0001**
	C	187.16 ± 18.00 a	169.94 ± 25.57 a	0.586
*Aphis gossypii*	E1	0.27 ± 0.27 a	7.93 ± 2.27 b	**<0.0001**
	E2	0 ± 0 a	159.40 ± 32.18 b	**<0.0001**
	Mean Duration of Waveform Events (min)			
	np	25.07 ± 4.11 a	9.39 ± 2.81 b	**<0.0001**
	C	15.51 ± 2.09 a	15.30 ± 4.59 a	0.563
	E1	2.18 a	3.01 ± 0.98 a	0.875
	E2	0 ± 0 a	98.82 ± 32.96 b	**<0.0001**
	Sequential ^a^ (min)			
	Start EPG—1st Probe	19.83 ± 2.32 a	3.57 ±1.36 b	**<0.0001**
	Start EPG—1st E	463.61 ± 16.38 a	175.84 ± 34.26 b	**<0.0001**

**^a^ Start EPG—1st Probe**: Time to 1st probe from the start of EPG; **Start EPG—1st E**: Time from start of EPG to 1st E. Means followed by the same letter, in the same row, do not differ significantly (*p* > 0.05) using Mann–Whitney U-test.

**Table 3:** Total duration of each waveform event (np, non-phloem phase, E1 and E2) as proportions of the time spent within the 8-h of EPG recording of *A. citricidus* and *A. gossypii*. Highlighted in bold those variables that showed striking significant differences between treatments.

Aphid Species	Aphid Behaviour *	Fruit Peduncles	Lime Leaves	*χ* ^2^	*p*
*Aphis citricidus*	Non-Probing	78.53 a	26.36 a	56.321	**<0.0001**
	Stylet Penetration (Non-phloem Phase)	21.47 a	33.25 a	3.653	0.585
	Salivation into Phloem (E1)	0 a	0.12 a	-	>0.999
	Phloem Ingestion (E2)	0 a	40.27 b	50.000	**<0.0001**
*Aphis gossypii*	Non-Probing	61.01 a	20.21 b	34.879	**<0.0001**
	Stylet Penetration (Non-phloem Phase)	38.94 a	44.92 a	0.739	0.395
	Salivation into Phloem (E1)	0.05 a	1.65 a	2.020	0.249
	Phloem Ingestion (E2)	0 a	33.21 b	39.521	**<0.0001**

* Variables followed by the same letter, in the same row, do not differ significantly (*p* > 0.05) according to the chi-square (*χ*^2^) test.
